# Portability of Polygenic Risk Scores for Sleep Duration, Insomnia and Chronotype in 33,493 Individuals

**DOI:** 10.3390/clockssleep5010002

**Published:** 2022-12-30

**Authors:** Anna Perkiö, Ilona Merikanto, Katri Kantojärvi, Tiina Paunio, Nasa Sinnott-Armstrong, Samuel E. Jones, Hanna M. Ollila

**Affiliations:** 1 Institute for Molecular Medicine Finland, Helsinki Institute of Life Science, University of Helsinki, 00290 Helsinki, Finland; 2 SleepWell Research Program, Faculty of Medicine, University of Helsinki, 00290 Helsinki, Finland; 3 Department of Public Health and Welfare, Finnish Institute for Health and Welfare, 00271 Helsinki, Finland; 4 Orton Orthopedics Hospital, 00280 Helsinki, Finland; 5 Department of Psychiatry, Faculty of Medicine, University Central Hospital, University of Helsinki, 00290 Helsinki, Finland; 6 Department of Genetics, Stanford University School of Medicine, Stanford, CA 94305, USA; 7 Broad Institute of MIT and Harvard, Cambridge, MA 02142, USA; 8 Center for Genomic Medicine, Massachusetts General Hospital, Boston, MA 02114, USA; 9 Anesthesia, Critical Care, and Pain Medicine, Massachusetts General Hospital and Harvard Medical School, Boston, MA 02114, USA

**Keywords:** circadian rhythms, eveningness, insomnia symptoms, morningness, polygenic score, PRS

## Abstract

Polygenic risk scores (PRSs) estimate genetic liability for diseases and traits. However, the portability of PRSs in sleep traits has remained elusive. We generated PRSs for self-reported insomnia, chronotype and sleep duration using summary data from genome-wide association studies (GWASs) performed in 350,000 to 697,000 European-ancestry individuals. We then projected the scores in two independent Finnish population cohorts (*N* = 33,493) and tested whether the PRSs were associated with their respective sleep traits. We observed that all the generated PRSs were associated with their corresponding traits (*p* < 0.05 in all cases). Furthermore, we found that there was a 22.2 min difference in reported sleep between the 5% tails of the PRS for sleep duration (*p* < 0.001). Our findings indicate that sleep-related PRSs show portability across cohorts. The findings also demonstrate that sleep measures using PRSs for sleep behaviors may provide useful instruments for testing disease and trait associations in cohorts where direct sleep parameters have not yet been measured.

## 1. Introduction

Sleep behaviors, such as insomnia, sleep duration and circadian type (chronotype), are all traits with a significant heritable component that can be captured from twin studies [1,2,3,4] and population-based GWAS studies alike, where individual genetic liability and variants are associated with these traits [5,6,7,8]. Many individual variants that associate with sleep traits are located at canonical circadian and neurotransmitter genes and in loci that overlap with those associated with the risk of cardiometabolic or psychiatric diseases [9]. At the same time, poor sleep quality, evening type, as well as both long and short sleep durations are associated with cardiometabolic traits and overall mortality [5,7,10,11,12]. 

Polygenic risk scores (PRSs) have the potential to become a practical tool for estimating an individual’s liability to a trait or disease, based on their genetic makeup [13]. Moreover, currently, the explanatory power of an individual PRS is usually small but the PRS can be used as a phenotype proxy to use in studies where sleep measures have not been assessed. Furthermore, PRSs can be used to elucidate genetic versus environmental correlates in such analyses. 

A PRS is calculated by summing the number of independent risk variants, which are commonly weighted by corresponding effect size estimates derived from a genome-wide association study (GWAS). The purpose of a GWAS is to identify variants that are associated with the trait or disease of interest. As the power and accuracy of a PRS depend on the overall effect size of the variants that are used, genome-wide PRS provides greater predictive power in an unbiased fashion compared to selecting candidate variants among established genetic associations [14]. However, it is important to determine how much explanatory power a PRS generated in one population has in another population, in order to understand the portability of these scores.

The goal of this study was to assess whether there is discernible predictive power in PRSs generated for self-reported insomnia, morning type, and sleep duration in a Finnish cohort, given that the variant effect size estimates originate from European-ancestry participants in the UK Biobank. In other words, we aimed to address the portability of sleep-related PRSs between two unrelated European populations.

**Figure 1:**
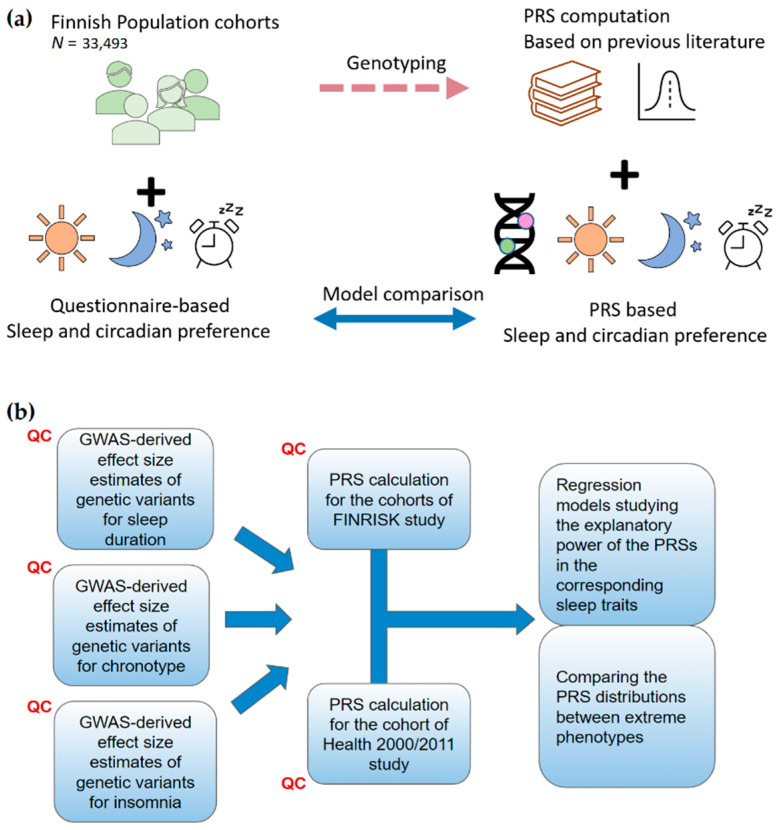
(**a**) A schematic diagram of the workflow of the study. (**b**) Analytical workflow, including quality control (QC) of the data.

In brief, the workflow of the study was to calculate the PRSs for each trait and for each participant of the utilized Finnish cohorts, based on the effect size estimates from the GWAS studies utilizing the UK Biobank cohort. Subsequently, the association between the scores and the corresponding traits was studied with regression models. Furthermore, the distributions of the PRSs for each trait were compared between the extreme, dichotomized phenotype groups. For further clarification of the utilized materials and methods, the summarized workflow of our study can be found also in Figure 1.

## 2. Results

### 2.1. Polygenic Risk Scores for the FINRISK Cohorts

We generated PRSs for self-reported insomnia, chronotype and sleep duration for 26,229 participants of the FINRISK study, which consists of Finnish adults aged 24 to 74 years. The scores for all three studied traits were associated with their corresponding continuous/categorical phenotypes (*p* < 0.001 for all traits, Table 1). *R*^2^, the coefficient of determination, indicates the proportion of the explained variance in the sleep trait. For the PRSs for morningness and sleep duration, the *R*^2^ was 0.021 and 0.004, respectively.

We tested the association of each PRS against measures of extreme dichotomized chronotype (absolutely “morning” people and absolutely “evening” people), short and long sleepers (people sleeping 6 h or less or 9 h or more, respectively) and insomnia (people with severe or no insomnia symptoms) using McFadden’s pseudo-*R^2^* (trait pseudo-*R^2^* = 0.033 and 0.010 and 0.003, respectively, Table 1). As with the continuous traits, the associations were significant across all traits (*p* < 0.001). While McFadden’s pseudo-*R^2^* is not directly comparable to the standard *R^2^*, our results demonstrate that the explanatory power of the PRS is higher in the extreme tails of the trait distributions. 

**Figure 2:**
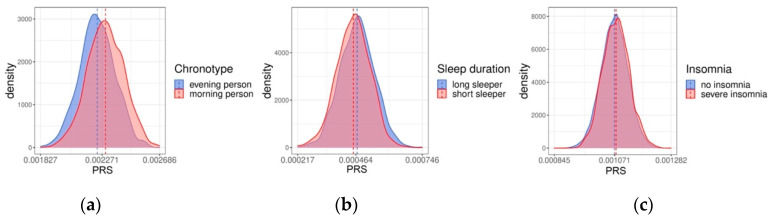
The distribution of polygenic risk scores (PRSs) for self-reported (**a**) morningness (**b**) sleep duration and (**c**) insomnia in FINRISK participants. The dashed lines mark the mean values for each group.

To further investigate the relationship between the PRSs and traits, the distributions of the PRSs in long sleepers vs. short sleepers, absolutely morning people vs. absolutely evening people, and people reporting no insomnia vs. people reporting severe insomnia were compared (Figure 2). We found a statistically significant difference between the long and short sleepers (*p* < 0.001), as well as between morning and evening people (*p* < 0.001). Furthermore, while individuals with no insomnia symptoms and severe insomnia symptoms had smaller difference between the absolute values in group means (0.00107 and 0.00108 with no insomnia and severe insomnia, respectively), the difference was again statistically significant, as supported by a *t*-test (*p* < 0.001).

Since previous studies have found that the power of a PRS is most prominent in the extreme tails of the score distribution [14,15], we were also interested to examine if there would be a difference in hours slept between individuals whose PRSs for sleep duration were in the 5% extreme tails of the score distribution. The difference in means between the two groups was 22.2 min. The participants in the top 5% PRS group slept 7 h 57.5 min, whereas the bottom 5% group slept 7 h 35.3 min, on average (*p* < 0.001). Even though the 22.2 min difference in means is prominent, there are still both long and short sleepers present in both tails (*SD* = 1 h 3.6 min and 1 h 3.0 min for high and low score tails, respectively).

### 2.2. Validation of the PRSs in Health 2000/2011 Cohort

**Figure 3:**
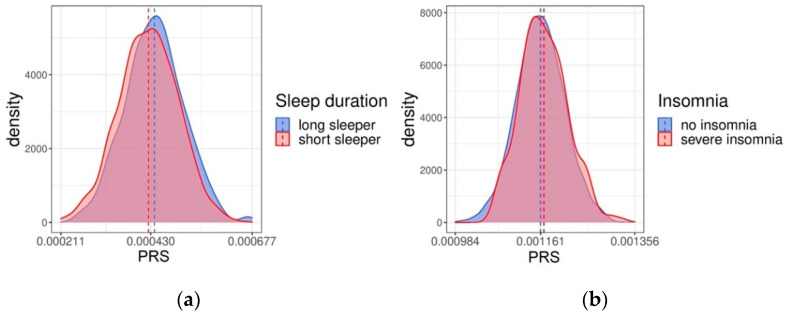
The distribution of polygenic risk scores (PRSs) for self-reported (**a**) sleep duration and (**b**) insomnia in Health 2000/2011 participants. The dashed lines mark the mean values for each group.

To further validate the results obtained from the FINRISK dataset, we replicated the analysis using the smaller cohort from Health 2000/2011 studies, which is an independent sample of Finns aged 18 to 99 (N = 7264). Chronotype information was not available for Health 2000/2011 cohort, so only sleep duration and insomnia were inspected with regression models (Table 2). For sleep duration, pseudo-*R^2^* was 0.008, and for insomnia 0.003. Both results were similar to what was found in FINRISK data, even though the explanatory power remained a little lower in Health 2000/2011. The distributions of the PRSs in short and long sleepers, as well as individuals with no self-reported insomnia and severe insomnia, are shown in Figure 3. The means between the groups visibly differ from each other for sleep duration *(p* < 0.001)*,* but again the difference is not as large for insomnia (*p* = 0.02). 

We again further examined the sleep durations of individuals in the top and bottom 5% PRS groups. Health 2000/2011 participants report sleeping less than FINRISK participants; the mean sleep duration of the top 5% PRS group was 7 h 33.6 min (SD = 1 h 1.1 min), whereas the bottom 5% average was 7 h 17.4 min (SD = 1 h 3.0 min). Hence, the difference between means was 16.2 min. The difference was smaller than that observed in FINRISK data, but nevertheless statistically supported (*p* = 0.001). 

## 3. Discussion

In this paper, we investigated the portability of polygenic risk scores between White European populations and cohorts, more specifically, between cohorts from the UK and Finland. We demonstrated that PRS for sleep duration, chronotype and insomnia show significant trait association and portability in two independent cohorts from a region independent of that where the genotypic effect sizes were originally calculated. Furthermore, we found that individuals at the top and bottom 5% of the PRS reported approximately 20 min difference in sleep duration, suggesting that PRS provides insight into sleep, particularly at the ends of the PRS distribution.

We discovered that while we found strong evidence of an association between the PRSs and the sleep traits, the overall explanatory power of habitual sleep duration, insomnia or chronotype was small at the level of the whole adult population. This finding is in agreement with earlier studies in the field of PRSs. Indeed, earlier literature shows that the PRSs usually have low explanatory power on the traits of interest, and even an *R^2^* of less than 1% are often reported [16,17]. In addition, it is important to note that genetic variants generally add up to around 10–50 percent of the phenotypic variance [14] and provide insight particularly on the genetic component of natural variation in sleep and individual circadian timing. Therefore, our findings indicate the largest benefit to understanding sleep and circadian typology as a phenomenon is achieved in cohorts where both genetic liability (through PRS) and the measured phenotypes are available, as was the case in the utilized dataset.

When it comes to the biological pathways associated with the studied sleep traits, the GWAS data utilized in this study have shown association as follows: for insomnia, there was enrichment of genes involved in ubiquitin-mediated proteolysis, as well as of genes expressed in multiple brain regions, skeletal muscle, and adrenal gland [7]. For chronotype, it was found that the genes expressed in the retina, hindbrain, hypothalamus, and pituitary, as well as those participating in the circadian regulation, cAMP, glutamate and insulin signaling pathways, were significantly enriched [6]. Furthermore, genes linked to striatum and subpallium development, mechanosensory response, dopamine binding, synaptic neurotransmission and plasticity were enriched for sleep duration [5]. It is likely that our PRSs also capture variability related to the functions of these same pathways. While the overall explanatory power of the PRSs was relatively low, it is to be highlighted that we showed an apparent difference at the very ends of the sleep duration PRS distribution, with individuals at the 5% ends of the distribution having ~20 min shorter sleep duration based on their PRS alone. Estimating a population average of 7.5 h of sleep, 20 min corresponds to 4.4% of total sleep time. This finding is nearly identical to the estimate from the original GWAS on sleep duration (22.2 min) [5] and emphasizes both the magnitude and the reproducibility of genetic risk scores. To put this into a clinical context, PRS are already informative for clinical endpoints, including cardiovascular and cancer risk prediction. For example, Mars et al. found that high PRS was associated both with the age of onset and the overall lifetime risk of coronary heart disease, type 2 diabetes, atrial fibrillation, breast cancer, as well as prostate cancer [18]. PRSs for different diseases and psychobehavioral traits have also been found to associate with the number of healthy life years lost either by worsened quality of life or premature death [15]. For instance, being in the top 10% of the multisite chronic pain PRS distribution accounted for over 3.5 healthy life years lost, when compared to the rest of the cohort (90%). Our own results again demonstrate the potential of the PRSs in risk prediction, especially in the extreme tails of the score distributions.

The portability of PRSs for different populations and ethnic groups has been an intense avenue of research, as it affects the possibility of obtaining the scores from bench to bedside. Multiple studies have reported that generalizing White European-derived risk estimates to other ethnicities, such as Black, Asian and Jewish people, caused a significant risk overestimation or poorer median effect sizes [19,20]. In contrast, some studies have reported good portability, for example between European and Asian or Hispanic cohorts [19,21,22]. In other words, it is yet not completely clear in which cases a particular PRS can be generalized in a particular population or cohort. Regardless, the statistically significant associations between our PRSes and the corresponding sleep traits demonstrated that the portability between the two different European populations was, in this case, robust. 

Our findings should be interpreted in the light of the following limitations; First, the current study may be reduced in the power to detect associations between our cohort and the UK Biobank, as the original questions may be interpreted slightly differently in different populations. Second, our cohort is smaller than the initial UK Biobank cohort and will have less precise estimates due to the smaller sample size. Third, it is unlikely that the phenotypic characterization perfectly reflects that of other populations, and therefore portability should be addressed separately in multiethnic settings.

In conclusion, our findings indicate an added value in collecting carefully phenotyped cohorts where different sleep measures can be assessed and used to understand the genetic and environmental determinants. Furthermore, the findings suggest direct utility of PRSs in cohorts where sleep measures have not yet been collected. Finally, PRSs for sleep can be used in cohorts where study participants cannot be contacted, and sleep measures cannot be collected.

## 4. Materials and Methods

### 4.1. GWAS Summary Statistics

GWAS summary statistics for sleep duration were obtained from a study by Dashti et al. [5], chronotype (morningness) summary statistics from Jones et al. [6], and insomnia (frequent vs. no insomnia symptoms) summary statistics from Lane et al. [7]. In all three studies, only participants with European ancestry were included. Observed genome-wide SNP-based heritability *(h^2^)* was 13.7%, 9.8% and 16.7% for chronotype, sleep duration and frequent insomnia symptoms, respectively. All the studies were based on UK Biobank data, described in detail elsewhere [23]. In summary, UK Biobank is a health resource of more than 500,000 people living in the United Kingdom. The participants, aged between 40 to 69, consented to partake in detailed anthropometric measures, self-report questionnaires and genotyping. Furthermore, these data were merged with the longitudinal health and medical records, yielding diverse phenotypic information. In addition to this cohort, Jones et al. utilized GWAS statistics from 23andMe cohort (described in full in [24]). It consists of customers of the personal genetics company 23andMe, Inc., who granted their data for research purposes. 

All three datasets were lifted over from hg19 to hg38, and only variants with imputation quality score (INFO) > 0.8 and minor allele frequency (MAF) > 0.01 were extracted. Furthermore, all multiallelic and ambiguous variants were excluded. Ultimately, 9,290,550; 11,343,186 and 11,343,186 variants remained from the chronotype, insomnia and sleep duration datasets, respectively. 

### 4.2. Target Data

Our data consisted of five independent FINRISK study cohorts 1992 (N = 7927), 1997 (N = 11,500), 2002 (N = 13,498), 2007 (N = 12,000) and 2012 (N = 10,000), as well as participants of Health 2000 (N = 9922) and Health 2011 (N = 11,051) studies.

FINRISK is a large-scale cross-sectional study based on the Finnish adult population (25 to 74 years), sampling from six selected areas across the country. It aimed to collect information on risk factors for different chronic illnesses. The same numbers of men and women across 10-year age groups were sampled from each geographical area. The participants were asked to fill in a mail survey, as well as to participate in a health examination at a local health care center, which included blood sampling. Furthermore, some of the blood samples were genotyped, yielding genotypic information for 30,625 participants from five areas (excluding Lapland). The study is explained in more detail elsewhere (see [25] and Finnish Institute of Health and Welfare BioBank: https://thl.fi/en/web/thl-biobank/for-researchers/sample-collections/the-national-finrisk-study-1992-2012, accessed on 20 July 2022).

The Health 2000 and 2011 studies aimed to assess health and welfare, as well as the related determining factors, in the Finnish adult population (18 to 99 years). The two cohorts are largely overlapping, since the participants of the Health 2000 study that were still alive were asked to participate again in 2011 (N = 8135). The data were collected using interviews, health examinations and questionnaires. In addition, genotype data were available for 7543 participants. For more detailed information about the study, see the Finnish institute for health and welfare webpage (https://thl.fi/en/web/thlfi-en/research-and-development/research-and-projects/health-2000-2011, accessed on 9 June 2022). 

DNA samples extracted from whole blood were genotyped with Illumina (Illumina Inc., San Diego, CA, USA) and Affymetrix arrays (Thermo Fisher Scientific, Santa Clara, CA, USA), depending on the batch. Genotypes were called using GenCall and zCall algorithms for Illumina, and AxiomGT1 algorithm for Affymetrix data. All the genotypes were then lifted over to build version 38 (GRCh38/hg38) following the protocol described here: dx.doi.org/10.17504/protocols.io.nqtddwn (accessed on 9 June 2022). Both the FINRISK and Health 2000/2011 datasets were imputed against the population-specific SISu v3 imputation reference panel (Pärn et al., manuscript in preparation). For a detailed imputation protocol, see: https://www.protocols.io/view/genotype-imputation-workflow-v3-0-e6nvw78dlmkj/v1?version_warning=no (accessed on 9 June 2022).

Health 2000/2011 and FINRISK genotype data were both filtered by excluding variants with imputation INFO < 0.9 and retaining only autosomal variants. In addition, we excluded individuals with substantially high or low heterozygosity estimates (i.e., for which the F coefficient was more than three standard deviation units from the mean), Lastly, all sample duplicates and first- and second-degree relatives were removed using PLINK 1.9 --rel-cutoff 0.125. As a result, FINRISK data consisted of 26,229 individuals and 12,547,122 variants, whereas Health 2000/2011 data had 7264 individuals and 12,049,664 variants. 

### 4.3. Assessing Sleep Traits

The studied sleep traits were assessed based on individuals’ questionnaire answers. The questions were asked either in Finnish or Swedish, as both are official languages in Finland. All questionnaire forms can also be found in English on the Finnish Institute of Health and Welfare web page (https://thl.fi/en/web/thlfi-en/research-and-development/research-and-projects/the-national-finrisk-study/questionnaires, accessed on 20 July 2022; https://thl.fi/en/web/thlfi-en/research-and-development/research-and-projects/health-2000-2011/forms, accessed on 20 July 2022). In short, chronotype was assessed only in FINRISK ‘07 and ‘12, with the question 19 from Morningness-Eveningness Questionnaire by Horne and Ostberg [26]. Essentially, this means that the participants were asked whether they identified themselves as more morning people or evening people, using a scale from 1 to 4 (1 absolutely a “morning” person–2 more “morning” person than “evening” person–3 more “evening” person than “morning” person–4 absolutely an “evening” person). Sleep duration was assessed by asking the average hours of sleep in 24 h from FINRISK cohorts ‘07, ‘12, and Health 2000/2011. Those participants that reported sleeping 3 h or less or 16 h or more were excluded from the analysis. For insomnia, FINRISK cohorts were asked the question “Do you suffer from insomnia”, with a scale from 1 to 3 (often–sometimes–not at all). For Health 2000 and 2011 cohorts, the insomnia symptom severity was assessed with a scale from 1 to 5 (from no symptoms to severe sleeplessness). The questions and their choice of responses can be found in detail in Table A1. 

Health 2011 was a follow-up to Health 2000; thus, 8135 of the participants were asked to fill in the same questionnaires twice. Therefore, one answer per participant per question was randomly selected for the analysis. 

### 4.4. Generating Risk Scores

PRSs were generated with PLINK 1.9 [27]. The imputed genotype data were first clumped, by only extracting independent variants with a *p* < 5 × 10^−4^, using a 250 kb window radius and a 0.1 threshold for *R*^2^. PRS was then calculated for the clumped set using PLINK’s default settings (i.e., the sum of an individual’s risk alleles, weighted by risk allele effect sizes).

### 4.5. Estimating the Explanatory Power of the Risk Scores

Estimates of the predictive power for the genetic scores were based on linear regression and logistic regression models, in which the dependent variable was the trait of interest, and the independent variables were the PRS, age, sex, and the first six principal components (PCs). The PCs were calculated based on the pruned variants to account for genetic structure. Genetic structure (or population stratification) is the presence of allelic differences within the target population, mainly caused by differences in ancestry [28]. Genetic structure is not a special feature of the Finns, but rather it can be detected in all populations of the world [29,30,31].

For the logistic models, the traits were dichotomized to produce binary variables. For chronotype, only the individuals who identified themselves as “Absolutely a morning person” or “Absolutely an evening person” were included in the model. For sleep duration, only people who reported sleeping 6 h or less (“short sleeper”) or 9 h or more (“long sleeper”) were extracted. For insomnia, people who reported having no insomnia symptoms, and people reporting “severe symptoms” (defined as answer “1” in FINRISK, and answer “4” or “5” in Health 2000/2011) were used to define the binary insomnia symptom phenotype and included in the logistic model. As the standard *R*^2^ cannot be calculated for logistic models, we estimated the predictive power with McFadden’s pseudo-*R*^2^ instead. The used formula for the metric was 1-d_1_/d_0_, where d_1_ is the deviance of the logistic model with PRS, and d_0_ is the deviance of the logistic model without PRS as a covariate.

## Figures and Tables

**Table 1:** Summary of the regression models for chronotype, sleep duration and insomnia based on FINRISK participants. Dependent and independent variables are sleep traits and PRS, respectively. PRS = polygenic risk score, p*R*^2^ = pseudo-*R*^2^.

	PRS in Linear Model			PRS in Logistic Model		
	N	*R* ^2^	*p*	N	p*R*^2^	*p*
Chronotype	8530	0.021	<0.001	2993	0.033	<0.001
Sleep duration	7647	0.004	<0.001	2016	0.010	<0.001
Insomnia	-	-	-	15,843	0.003	<0.001

**Table 2:** Summary of the regression models for sleep duration and insomnia based on Health 2000/2011 participants. Dependent and independent variables are sleep traits and PRS, respectively. PRS = polygenic risk score, p*R*^2^ = pseudo-*R*^2^.

	PRS in Linear Model			PRS in Logistic Model		
	N	*R* ^2^	*p*	N	p*R*^2^	*p*
Sleep duration	6389	0.004	<0.001	1812	0.008	<0.001
Insomnia	-	-	-	3335	0.003	0.03

## Data Availability

Data was obtained from The Finnish institute for Health and Welfare (THL) and are available from https://thl.fi/en/web/thlfi-en (accessed on 20 July 2022) with the permission of THL.

## Appendix A

**Table A1:** Questions utilized to determine sleep duration, chronotype and insomnia of FINRISK and Health 2000/2011 cohorts. The full questionnaires can be found in the Finnish Institute for Health and Welfare webpage (both in English and in Finnish): https://thl.fi/en/web/thlfi-en/research-and-development/research-and-projects/the-national-finrisk-study/questionnaires, accessed on 20 July 2022; https://thl.fi/en/web/thlfi-en/research-and-development/research-and-projects/health-2000-2011/forms, accessed on 20 July 2022.

	Sleep Duration	Chronotype	Insomnia
FINRISK ‘92	unavailable	unavailable	Do you suffer from insomnia? → 1 (Not at all) → 2 (Sometimes) → 3 (Often)
FINRISK ‘97	unavailable	unavailable	Do you suffer from insomnia? → 1 (Often) → 2 (Sometimes) → 3 (Not at all)
FINRISK ‘02	unavailable	unavailable	Do you suffer from insomnia? → 1 (Often) → 2 (Sometimes) → 3 (Not at all)
FINRISK ‘07	How many hours on average do you sleep in 24 h? → ___ hours + ___ mins	There are so-called “morning people” (early to rise, early to bed) and “evening people” (late to rise, late to bed). Which are you? → 1 absolutely a “morning person” → 2 more “morning” than “evening person” → 3 more “evening” than “morning person” → 4 absolutely an “evening person”	Do you suffer from insomnia? → 1 (Often) → 2 (Sometimes) → 3 (Not at all)
FINRISK ’12	How many hours on average do you sleep in 24 h? → ___ hours + ___mins	There are so-called “morning people” (early to rise, early to bed) and “evening people” (late to rise, late to bed). Which are you? → 1 absolutely a “morning person” → 2 more “morning” than “evening person” → 3 more “evening” than “morning person” → 4 absolutely an “evening person”	Do you suffer from insomnia? → 1 (Often) → 2 (Sometimes) → 3 (Not at all)
Health 2000	How many hours on average do you sleep in 24 h? → ___ hours	unavailable	Sleeping → 1 I am able to sleep normally, i.e., I have no problems with sleeping → 2 I have slight problems with sleeping, e.g., difficulty in falling asleep, or sometimes waking at night → 3 I have moderate problems with sleeping, e.g., disturbed sleep, or feeling I have not slept enough → 4 I have great problems with sleeping, e.g., having to use sleeping pills often or routinely, or usually waking at night and/or too early in the morning → 5 I suffer from severe sleeplessness, e.g., sleep is almost impossible even with full use of sleeping pills or staying awake most of the night
Health 2011	How many hours on average do you sleep in 24 h? → ___ hours	unavailable	Sleeping → 1 I am able to sleep normally, i.e., I have no problems with sleeping → 2 I have slight problems with sleeping, e.g., difficulty in falling asleep, or sometimes waking at night → 3 I have moderate problems with sleeping, e.g., disturbed sleep, or feeling I have not slept enough → 4 I have great problems with sleeping, e.g., having to use sleeping pills often or routinely, or usually waking at night and/or too early in the morning → 5 I suffer from severe sleeplessness, e.g., sleep is almost impossible even with full use of sleeping pills or staying awake most of the night
